# From therapeutic to aesthetic value in education/art/occupational therapy: creative potential and tactile value

**DOI:** 10.1192/j.eurpsy.2025.1288

**Published:** 2025-08-26

**Authors:** E. Chirila

**Affiliations:** 1 omplex de Servicii Sociale Comunitare Pentru Copii și Adulţi, CONSILIUL JUDEŢEAN CLUJ DIRECŢIA GENERALĂ DE ASISTENŢĂ SOCIALĂ ŞI PROTECŢIA COPILULUI CENTRUL COMUNITAR JUDEŢEAN, Cluj-Napoca, Romania

## Abstract

**Introduction:**

Fingerprints multiply man in space and time. Educating man with his hand, he learns how to master spatial extent, weight, density, proportion, organizes the activity in order to experience the action. The beneficiary creates a unique universe in which he leaves his mark everywhere. Ergonomics is considered as the “science of work”, having the “man-work” relationship as its object of study. In art therapy, occupational and play therapy, which is based on traditional aesthetics, we talk about “sublimation”. (ChirilăE & Câmpean & Drăgan-Chirilă.D, 2021- 10-13 April)

**Objectives:**

By means specific to the visual arts, hidden, overdue individual skills are detected, which bring the beneficiary closer to normality, to real life. We can observe that all things in nature are shapes modeled in volumes made up of fillings and voids, which constantly transform the appearance of the world. All these angles are synthesized in the brain, in order to understand the three-dimensionality of the environment as a unitary whole. This happens when we are in a position to be aware, actively, of the deep relationships that the environment implies, in general.

**Methods:**

Tactile value is the origin of any act of creation, the hand removes the sense of touch from receptive passivity. The evaluation of fine and gross motor skills is done by modeling, we use the sense of touch, we follow the use of hands during creation. In the testing activities through product analysis we use the visual language, that of the symbolic language of anthropomorphism. Holistic training is done on the potter’s wheel or through 3D multimedia installations. Creating works of art, artefacts, or using arts-based jobs are test activities for art therapy, occupational therapy and ergotherapy, which provide us with information about the author with the help of images and shapes. Each beneficiary can create the images themselves, with which they want interact or can use arts materials and games according to his needs of expression.

**Results:**

These activities allow the beneficiaries to reach a certain level of introspection and to “work through” their problems in a constructive manner. The activities can be approached in several distinct groups of people, who can be qualified in different artistic professions such as: visual artist, decorator, designer, they can practice adaptive-”raw arts”. (Chirila, E 2011, PhD Thesis p 398)

**Image 1:**

**Figure:**
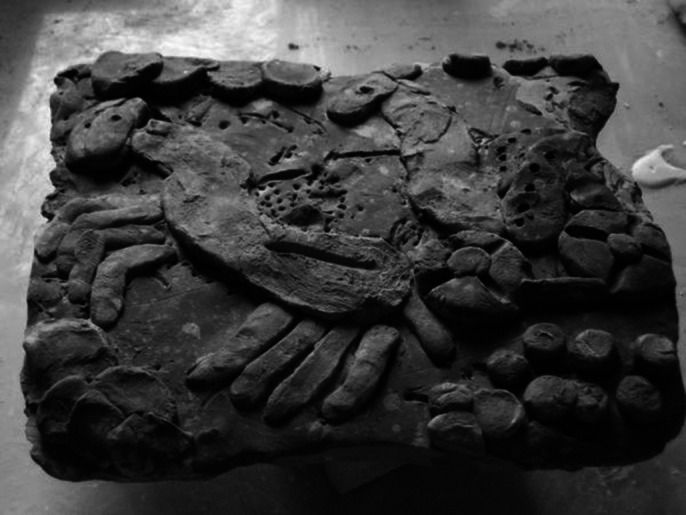

**Image 2:**

**Figure:**
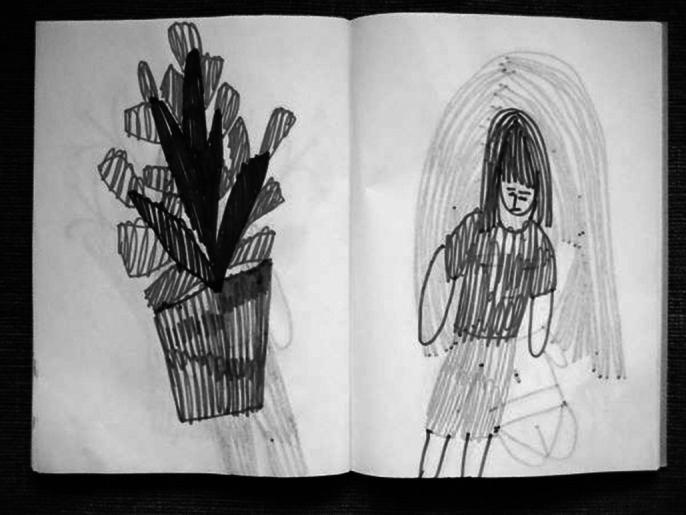

**Image 3:**

**Figure:**
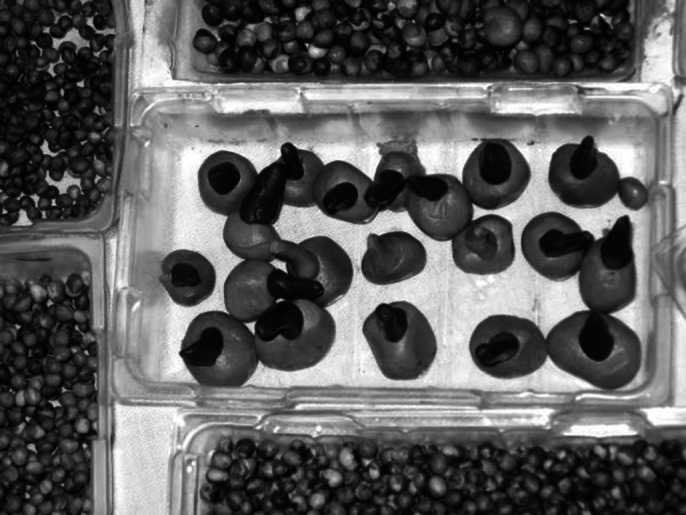

**Conclusions:**

The foundations are laid for an occupational therapy workshop for adults with special needs. In these types of activities, the beneficiaries who do not have artistic skills can learn useful skills for CES persons, who, to pursue a productive goal like “Work for the disabled”. (Chirila, E 2018 p50)

**Disclosure of Interest:**

E. Chirila Consultant of: NO

